# Differences in thromboelastometry parameters A10EXTEM and A10FIBTEM –as independent risk factors for mortality during bleeding

**DOI:** 10.1007/s00423-026-04104-4

**Published:** 2026-06-18

**Authors:** Hagen Bomberg, Theresa Mokry, Neeti Sharma, Klaus Görlinger, Stefan Wagenpfeil, Thomas Volk, Sven Oliver Schneider

**Affiliations:** 1 Department of Anaesthesiology, Intensive Care Medicine and Pain Medicine, Balgrist University Hospital, University of Zurich, Forchstrasse 370, Zurich, 8008 Switzerland; 2 Department of Anaesthesiology, Intensive Care Medicine and Pain Medicine, Saarland University Medical centre, Homburg/Saar, Germany; 3 Outcomes Research Consortium, UTHealth, Houston, TX USA; 4 Medical Director, Tem Innovations GmbH, Munich, Germany; 5 Institute for Medical Biometry, Epidemiology and Medical Informatics, Saarland University Medical centre, Saarland University, Homburg/Saar, Germany

**Keywords:** Mortality, Risk factor, Bleeding, Rotational thromboelastometry

## Abstract

**Purpose:**

During bleeding, the decrease A10_EXTEM_ and A10_FIBTEM_ (the clot firmness at 10 min) measured by rotational thromboelastometry (Rotem) can detect alterations in the fibrin contribution to clot firmness (FIBTEM) and in the extrinsic pathway (EXTEM). However, the significance of a decrease of A10_FIBTEM_ and A10_EXTEM_ for risk stratification in patients with bleeding remains unclear.

**Methods:**

1942 consecutive patients were retrospectively examined between 2014 and 2020. All patients were tested with Rotem during a hemorrhage at Saarland University Hospital. A10_EXTEM_ and A10_FIBTEM_ and their association with mortality at 30 days were tested using C statistic. A threshold value for A10_EXTEM_ reaching a specificity > 90% for 30-day mortality was selected and for A10_FIBTEM_ was fixed at <6 mm. Adjusted hazard ratios (adjHR [95% confidence interval]) were calculated with multivariable Cox models.

**Results:**

A10_EXTEM_ (C statistic 0.57, selected threshold≤39 mm) had a predictive power for 30-day mortality, whereas A10_FIBTEM_ did not. The 30-day mortality rate was significantly increased in patients with A10_EXTEM_≤39 mm (A10_EXTEM_≤39 mm: 32% versus A10_EXTEM_>39 mm: 16%, *p* < 0.001) and A10_FIBTEM_<6 mm (A10_FIBTEM_<6 mm: 25% versus A10_FIBTEM_≥6 mm: 18%, *p* = 0.03). The selected threshold values of A10_EXTEM_≤39 mm (adjHR 1.7 [1.3–2.3], *p* < 0.001) and A10_FIBTEM_<6 mm (adjHR 1.6 [1.1–2.2], *p* = 0.009) remained independent risk predictors for 30-day mortality even after adjustment for confounding factors. The leading cause of death in these populations were uncontrolled bleeding. Patients with A10_FIBTEM_<6 mm had significant more fibrinogen administration (A10_FIBTEM_<6 mm: 2.7 ± 3.7 g versus A10_FIBTEM_≥6 mm: 0.5 ± 1.6 g, *p* < 0.001).

**Conclusion:**

Our results indicate that A10_EXTEM_-detected alterations in the extrinsic pathway might be independent predictors for 30-day mortality in patients with bleeding. Nevertheless, A10_FIBTEM_ measurements to optimize treatment with fibrinogen is required.

**Supplementary Information:**

The online version contains supplementary material available at https://doi.org/10.1007/s00423-026-04104-4.

## Introduction

 Severe bleeding can impair coagulation by reducing clotting factors, potentially causing hemostatic failure. Rotational thromboelastometry (Rotem) provides a bedside diagnostic tool to identify such deficits quickly [1, 2].

Rotem is a whole-blood, point-of-care viscoelastic testing system that employs single-use reagents to perform distinct assays: EXTEM (tissue factor–activated extrinsic pathway activation without heparin neutralization), FIBTEM (fibrin contribution to clot firmness), INTEM (intrinsic pathway without heparin neutralization), and APTEM (extrinsic pathway with fibrinolysis inhibition). Together, the assays allow a differentiated assessment of coagulation dynamics, including early clot strength as reflected by the 10 min clot amplitude (A10) [3].

Ideally, treatment decisions regarding coagulation are guided by the specific Rotem assay results. Allowing target-based therapy on the identified deficit. Based on clot firmness at 10 min (A10) in EXTEM and FIBTEM, different treatment strategies are recommended: An isolated reduction of A10 EXTEM below 40 might be an indicator for platelet deficiency. An additional reduction of FIBTEM A10 below 10 mm suggests fibrinogen and platelet deficiency. Thus, it guides the use of when to treat with fibrinogen, platelets, or desmopressin [2].

Our aim was therefore to investigate the relationship between A10_EXTEM_, A10_FIBTEM_, and 30-day mortality. Our hypothesis was that there is an independent association between A10_EXTEM_, A10_FIBTEM_, and 30-day mortality in patients with bleeding.

## Materials and methods

The study was registered in the German Clinical Trials registration (registration number DRKS00026153) in September 2021 (principal investigator: PD Dr. med. Sven Oliver Schneider, Saarland University, Department of Anaesthesiology, Intensive Care Medicine and Pain Medicine, Homburg/Saar, Germany). After approval of the Ethical Committee (Ärztekammer Saarland; number 93/20), the data were retrospectively collected. The requirement for written informed consent was waived by the Ethical Committee. The data set in this study originates from the bleeding database at Saarland University Hospital. Previously, publications have presented data from the database in relation to CT_EXTEM_ and CT_INTEM_ [4, 5].

Bleeding episodes were identified based on the treating physician’s clinical judgement. Administration of coagulation factors followed a rotational thromboelastometry-guided bleeding management protocol and was tailored to the clinical situation. All patients were treated according to the protocol by Weber et al., which remained unchanged throughout the study period (2014–2020) [2]. Rotational thromboelastometry testing was routinely performed in all patients with bleeding and suspected coagulation abnormalities.

### Inclusion/exclusion criteria

All patients tested in the Saarland University Hospital by rotational thromboelastometry between 2014 and 2020 were included. Rotational thromboelastometry is available for all patients with bleeding and/or coagulation disorders in the Saarland University Hospital.

The study population was excluded in cases of missing measurements for CT_EXTEM_, A10_EXTEM_, CT_INTEM_, A10_FIBTEM_, CT_APTEM,_ A10_APTEM_, and missing information on outcome data, population characteristics, and comorbidities covariate data, or implausible values.

### Data source

Rotational thromboelastometry data are recorded during patient care and stored in the hospital’s medical reports and the bleeding database. Detailed information about the medical conditions of patients, along with the procedure and postoperative course, was extracted from the hospital database. All data were included in one registry.

### Rotational thromboelastometry analyses

The rotational thromboelastometry analyses (Rotem) were performed using the Rotem delta^®^ (Tem Innovations, Munich, Germany) and comprised measurements of EXTEM (tissue factor–activated extrinsic pathway activation without heparin neutralization), FIBTEM (fibrin contribution to clot firmness), INTEM (intrinsic pathway without heparin neutralization), and APTEM (extrinsic pathway with fibrinolysis inhibition). Clotting time (CT) and amplitude of clot firmness 10 min after CT = the clot firmness at 10 min (A10) were assessed for all assays. Each patient was considered only once. If more than one Rotem analysis was done in one patient, only the first Rotem analysis was considered.

### Proof of plausibility

Data integrity was evaluated according to specific rules to delete erroneously entered data and cases with missing information (proof of plausibility, Appendix 1).

### Missing data handling/sensitivity analyses

Patients with missing data were excluded from the analysis. Several sensitivity analyses were performed.

### Definition of the outcomes

The primary outcome was overall survival with a follow-up period of 30 days.

The secondary outcomes were: Severe adverse events were defined by the occurrence of pneumonia, pulmonary embolism, acute myocardial infarction, embolic apoplexy, peripheral arterial embolism and thrombosis, and gastrointestinal ischemia. Gastrointestinal ischemia was defined as occlusive or non-occlusive mesenteric ischemia, with the diagnosis confirmed by laparotomy, computed tomography, or mesenteric angiography.

### Data analysis

Continuous variables are expressed as means +/- standard deviations and were compared using Student´s t-test. Categorical variables are presented as absolute and relative frequencies and were compared with chi^2^ tests.

Receiver operating characteristic curves (C-statistic) were constructed to evaluate the predictive power of A10_EXTEM_ and A10_FIBTEM_ for the occurrence of 30-day mortality. The Youden Index was used to calculate the optimal threshold for CT_EXTEM_ in the prediction of 30-day mortality. The threshold value for A10_EXTEM_ reaching a specificity > 90% for 30-day mortality was selected. The threshold value for A10_FIBTEM_ was fixed at < 6 mm following clinical experience. The number needed to screen was calculated to measure how many patients must be screened with values A10_EXTEM_≤39 mm and A10_FIBTEM_<6 mm to avoid one death. The positive predictive value was defined as the number of true positives/(number of true positives + number of false positives). The negative predictive value was defined as the number of true negatives/(number of true negatives + number of false negatives).

Survival rates were estimated using the Kaplan-Meier method and compared using the log-rank test. Cox proportional hazards models were used to adjust for confounding factors, including all population characteristics and comorbidities. Pairwise dependent variable constellations with Pearson or Spearman correlation coefficients exceeding + 0.4 or less than − 0.4 were a priori specified as interaction terms in multivariable analyses to account for the issue of multicollinearity. Sensitivity analyses were performed, including confounding factors listed in Tables 1 and 2. Data analysis was performed using SPSS Statistics 29™ (IBM, Ehningen, Germany). Two-sided p-values ˂ 0.05 were considered statistically significant.

**Table 1:** Population characteristics, comorbidities and rotational thromboelastometry analysis

	A10 EXTEM > 39 mm		A10 EXTEM ≤39 mm		p-value	A10 FIBTEM ≥ 6 mm		A10 FIBTEM <6 mm		p-value
	(*n* = 1690)		(*n* = 252)			(*n* = 1764)		(*n* = 178)		
*Hospital admission*										
*Age (yrs)*	63	± 17	61	± 17	*0.1*	63	± 17	58	± 20	***< 0.001***
*Male (%)*	1111	(66)	140	(56)	***0.002***	1137	(64)	114	(64)	*0.9*
*Body mass index (kg/m* ^*2*^ *)*	27	± 6	27	± 6	*0.2*	27	± 6	26	± 5	***0.007***
*Emergency*	522	(31)	86	(34)	*0.3*	552	(31)	56	(31)	*1*
*Re-operation after post-operative bleeding*	374	(22)	81	(32)	***0.001***	406	(23)	49	(28)	*0.2*
*Department*										
*Cardiothoracic surgery with CPB*	654	(39)	45	(18)	***< 0.001***	658	(37)	41	(23)	***< 0.001***
*Trauma*	175	(10)	17	(7)	*0.1*	171	(10)	21	(12)	*0.4*
*General surgery (without liver)*	253	(15)	50	(20)	*0.05*	276	(16)	27	(15)	*0.9*
*Liver surgery*	110	(7)	37	(15)	***< 0.001***	128	(7)	19	(11)	*0.1*
*Thoracic surgery (without CPB)*	65	(4)	4	(2)	*0.1*	66	(4)	3	(2)	*0.2*
*Vascular Surgery (without CPB)*	73	(4)	15	(6)	*0.2*	76	(4)	12	(7)	*0.1*
*Gynecology (without obstetrics)*	38	(2)	12	(5)	***0.02***	43	(2)	7	(4)	*0.2*
*Gynecology (only obstetrics)*	21	(1)	12	(5)	***< 0.001***	27	(2)	6	(3)	*0.1*
*Urology*	59	(4)	11	(3)	*0.5*	62	(4)	8	(4)	*0.5*
*Neurosurgery*	69	(4)	7	(3)	*0.3*	70	(4)	6	(3)	*0.7*
*Pediatric (age < 14 years)*	23	(1)	5	(2)	*0.4*	20	(1)	8	(4)	***< 0.001***
*Other departments*	150	(9)	37	(15)	***0.004***	167	(9)	20	(11)	*0.4*
*Comorbidities*										
*Coronary heart disease*	487	(29)	39	(15)	***< 0.001***	500	(28)	26	(15)	***< 0.001***
*Cardiac insufficiency (EF < 45%)*	124	(7)	9	(4)	***0.03***	129	(7)	4	(2)	***0.008***
*Chronic obstructive pulmonary disease*	146	(9)	26	(10)	*0.4*	157	(9)	15	(8)	*1*
*Insulin-dependent diabetes mellitus*	110	(7)	16	(6)	*1*	115	(7)	11	(6)	*1*
*Liver disease Child C*	52	(3)	38	(15)	***< 0.001***	71	(4)	19	(11)	***< 0.001***
*Coagulation disorders*	160	(9)	62	(25)	***< 0.001***	194	(11)	28	(16)	*0.1*
*Anticoagulation prior Rotem analysis*										
*Aspirin 100mg*	442	(26)	32	(13)	***< 0.001***	443	(25)	31	(17)	***0.02***
*High molecular weight heparin*	255	(15)	42	(17)	*0.5*	269	(15)	28	(16)	*0.8*
*Low molecular weight heparin*	163	(10)	22	(9)	*0.7*	163	(9)	22	(12)	*0.2*
*Other anticoagulation*	128	(8)	14	(6)	*0.3*	136	(8)	6	(3)	***0.03***
*Rotational thromboelastometry*										
*CT EXTEM (s)*	75	± 38	118	± 146	***< 0.001***	78	± 56	111	± 120	***< 0.001***
*CT INTEM (s)*	216	± 109	271	± 185	***< 0.001***	220	± 121	252	± 142	***< 0.001***
*A10 EXTEM (mm)*	57	± 9	31	± 8	***< 0.001***	55	± 11	35	± 10	***0.001***
*A10 FIBTEM (mm)*	17	± 10	7	± 6	***< 0.001***	17	± 10	4	± 1	***< 0.001***
*CT APTEM (s)*	75	± 43	112	± 151	***< 0.001***	76	± 50	116	± 159	***< 0.001***
*A10 APTEM (mm)*	56	± 10	31	± 8	***< 0.001***	54	± 11	35	± 9	***< 0.001***

Population characteristics, Comorbidities, and Rotational thromboelastometry analysis. A10_EXTEM_>39 mm versus A10_EXTEM_≤39 mm. A10_FIBTEM_≥6 mm versus A10_FIBTEM_<6 mm. Continuous variables are expressed as mean and standard deviation and were compared with a t-test. Categorical variables are presented as numbers (column percentages) and were compared with chi^2^ tests. A10: amplitude of clot firmness 10 min after CT in mm. CPB: cardiopulmonary bypass. CT: clotting time in seconds (s). EF: ejection fraction. Rotem: Rotational thromboelastometry. APTEM: extrinsic pathway with inhibition of fibrinolysis. EXTEM: extrinsic pathway. FIBTEM: fibrin contribution to clot firmness. INTEM: intrinsic pathway without heparin neutralization. The leading department is selected in the departments. Cardiothoracic surgery with CPB includes patients undergoing surgery with cardiopulmonary bypass. Trauma includes traumatically injured patients, and patients suffering from multiple injuries that involve multiple organs. Other departments includes orthopedic surgery, ear, nose, throat, and maxillofacial surgery, and internal medicine. Pediatric includes patients with age < 14 years (10 patients had an age < 1 year). Emergency at hospital admission is defined as a surgical intervention necessary within 6 h after hospital admission. Coagulation disorders: known bleeding coagulation disorder, e.g., thrombocytopenia, factor deficiency, von Willebrand disease. Anticoagulation before the Rotem analysis includes anticoagulation in the hospital ward or at home. Other anticoagulants: Argatroban, Phenprocoumon, Factor Xa inhibitor, Dabigatran, and P2Y12 inhibitors. All significant values are marked in bold

**Table 2:** Outcome

	Before rotem analysis									
	A10 EXTEM > 39 mm		A10 EXTEM ≤39 mm		p-value	A10 FIBTEM ≥ 6 mm		A10 FIBTEM <6 mm		p-value
	(*n* = 1690)		(*n* = 252)			(*n* = 1764)		(*n* = 178)		
*Mech. ventilated (h)*	23	± 132	12	± 43	*0.2*	23	± 130	10	± 40	*0.2*
*Renal replacement therapy (%)*	192	(11)	43	(17)	***0.01***	216	(12)	19	(11)	*0.6*
*Length of stay (days)*										
*In ICU*	3	± 10	2	± 6	*0.5*	3	± 10	2	± 5	*0.2*
*In hospital*	7	± 15	8	± 14	*0.5*	7	± 15	6	± 11	*0.4*
*30-day mortality (%)*	*─*		*─*			*─*		*─*		*─*
*Packed red blood cells (n)*	2	± 6	4	± 7	***< 0.001***	2	± 6	2	± 4	*0.9*
*Platelet concentrate (n)*	1	± 2	2	± 5	***< 0.001***	1	± 3	1	± 2	*0.5*
*Fresh frozen plasma (n)*	1	± 4	1	± 3	*0.2*	1	± 4	1	± 3	*0.6*
*Prothrombin concentrate (IU)*	460	± 1660	1077	± 3007	***< 0.001***	520	± 1806	733	± 2658	*0.2*
*Fibrinogen (g)*	0.4	± 1.8	1.7	± 5.4	***< 0.001***	0.6	± 2.5	1.0	± 3.8	***0.02***
*Recombinant Factor VIIa (IU)*	2	± 37	5	± 65	*0.3*	2	± 44	0	± 0	*0.5*
*Factor XIII (IU)*	29	± 359	45	± 322	*0.5*	31	± 367	28	± 186	*0.9*
*Adverse events*										
*Pneumonia (%)*	150	(9)	27	(11)	*0.3*	161	(9)	16	(9)	*1*
*Pulmonary embolism (%)*	30	(2)	8	(3)	*0.1*	35	(2)	3	(2)	*1*
*Acute myocardial infarction (%)*	77	(5)	4	(2)	***0.03***	78	(4)	3	(2)	*0.1*
*Embolic apoplexy (%)*	57	(3)	5	(2)	*0.3*	58	(3)	4	(2)	*0.7*
*Peripheral arterial embolism and thrombosis (%)*	57	(3)	9	(4)	*0.9*	59	(3)	7	(4)	*0.7*
*Gastrointestinal ischemia (%)*	58	(3)	15	(6)	*0.1*	67	(4)	6	(3)	*1*
*Gastrointestinal bleeding (%)*	94	(6)	26	(10)	***0.007***	106	(6)	14	(8)	*0.3*
	**After rotem analysis**									
	**A10 EXTEM > 39 mm**		**A10 EXTEM ≤39 mm**		**p-value**	**A10 FIBTEM ≥ 6 mm**		**A10 FIBTEM <6 mm**		**p-value**
	**(** ***n*** ** = 1690)**		**(** ***n*** ** = 252)**			**(** ***n*** ** = 1764)**		**(** ***n*** ** = 178)**		
*Mech. ventilated (h)*	66	± 177	40	± 144	***0.03***	66	± 179	31	± 91	***0.01***
*Renal replacement therapy (%)*	329	(19)	57	(23)	*0.2*	357	(20)	29	(16)	*0.2*
*Length of stay (days)*										
*In ICU*	10	± 18	9	± 13	*0.7*	10	± 18	10	± 14	*0.2*
*In hospital*	22	± 33	20	± 25	*0.4*	22	± 33	21	± 26	*0.8*
*30-day mortality (%)*	274	(16)	80	(32)	***< 0.001***	310	(18)	44	(25)	***0.03***
*Packed red blood cells (n)*	4	± 6	3	± 5	*0.2*	4	± 6	3	± 4	*0.1*
*Platelet concentrate (n)*	1	± 2	1	± 2	*0.2*	1	± 2	1	± 2	*0.9*
*Fresh frozen plasma (n)*	1	± 6	4	± 47	***0.02***	1	± 19	1	± 2	*0.7*
*Prothrombin concentrate (IU)*	454	± 1447	865	± 1818	***< 0.001***	468	± 1462	904	± 1840	***< 0.001***
*Fibrinogen (g)*	0.5	± 1.6	2.1	± 3.4	***< 0.001***	0.5	± 1.6	2.7	± 3.7	***< 0.001***
*Recombinant Factor VIIa (IU)*	27	± 921	8	± 51	*0.7*	26	± 902	4	± 36	*0.7*
*Factor XIII (IU)*	61	± 1020	64	± 373	*0.9*	60	± 1000	77	± 402	*0.8*
*Adverse events*										
*Pneumonia (%)*	184	(11)	12	(5)	***0.002***	183	(10)	13	(7)	*0.2*
*Pulmonary embolism (%)*	15	(1)	2	(1)	*1*	16	(1)	1	(1)	*1*
*Acute myocardial infarction (%)*	10	(1)	2	(1)	*0.7*	11	(1)	1	(1)	*1*
*Embolic apoplexy (%)*	26	(2)	4	(2)	*1*	26	(1)	4	(2)	*0.3*
*Peripheral arterial embolism and thrombosis (%)*	11	(1)	1	(0)	*1*	12	(1)	0	(0)	*0.6*
*Gastrointestinal ischemia (%)*	47	(3)	4	(2)	*0.4*	47	(3)	4	(2)	*1*
*Gastrointestinal bleeding (%)*	10	(1)	5	(2)	***0.04***	13	(1)	2	(1)	*0.6*

Outcome related to A10_EXTEM_ and A10_FIBTEM_. A10_EXTEM_>39 mm versus A10_EXTEM_≤39 mm. A10_FIBTEM_≥6 mm versus A10_FIBTEM_<6 mm. The outcome was split in the time before and after the rotational thromboelastometry analysis. Continuous variables are expressed as mean and standard deviation and were compared with a t-test. Categorical variables are presented as numbers (column percentages) and were compared with chi^2^ tests. A10: amplitude of clot firmness 10 min after CT in mm. CT: clotting time. EXTEM: extrinsic pathway. FIBTEM: fibrin contribution to clot firmness. ICU: intensive care unit. Rotem: Rotational thromboelastometry. Packed red blood cells (450mL), Platelet concentrate (270mL), Fresh frozen plasma (300mL). All significant values are marked in bold

## Results

During the study period, 2656 patients had documented multiple analyses. Among these, 467 patients had no A10_EXTEM_ or A10_FIBTEM_ analysis, 86 patients had missing information about outcome, comorbidities, or implausible data, and 161 patients had missing information about CT_EXTEM_, CT_INTEM_, CT_APTEM_, and A10_APTEM_. The final study population includes 1942 patients.

Patients with A10_EXTEM_≤39 mm showed more bleeding after surgery, whereas patients with A10_FIBTEM_<6 mm were younger and had less cardiothoracic surgery with cardiopulmonary bypass (Table 1). Patients with lower A10_EXTEM_≤39 mm and A10_FIBTEM_<6 mm had more pathological findings in the rotational thromboelastometry analyses (Rotem) (Table 1).

**Fig. 1:**
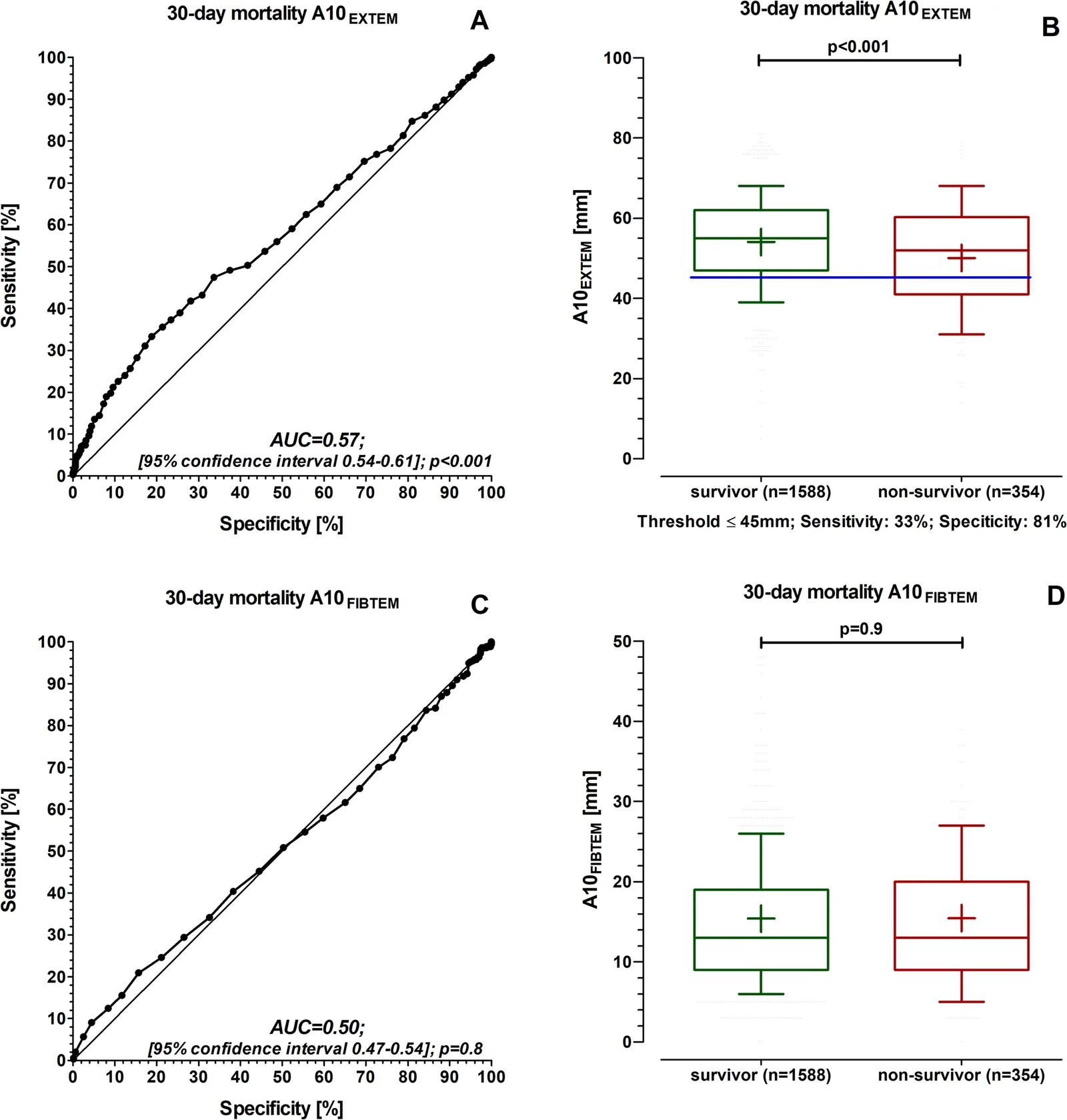
Receiver operating characteristic curves (ROC) were constructed (C-statistic) to evaluate the predictive power of A10_EXTEM_ (**A**) and A10_FIBTEM_ (**C**) for 30-day mortality. Box and whisker plot with the 10th and 90th percentiles for A10_EXTEM_ (**B**) and A10_FIBTEM_ (**D**). Solid dash: median values. The plus symbol: mean values. Blue solid dashed line: optimal threshold. AUC: area-under-the-curve. The Youden Index was used to calculate the optimal threshold for A10_EXTEM_ in the prediction of 30-day. Data were collected in real time by the attending physician or nurse in parallel to the patient`s treatment. Each patient was considered only once. If more than one Rotational thromboelastometry analysis was done in one patient, only the first Rotem analysis was considered. A10: amplitude of clot firmness 10 min after CT in mm. CT: clotting time. EXTEM: extrinsic pathway. FIBTEM: fibrin contribution to clot firmness

C-statistic showed that A10_EXTEM_ had discrimination for prediction of 30-day mortality (optimal threshold ≤ 45 mm, C-statistic 0.57, *p* < 0.001, sensitivity 33% and specificity 81%; Fig. 1), in contrast, not for A10_FIBTEM_. The number needed to screen (NNS), the positive predictive value (PPV), and the negative predictive value (NPV) were calculated for 30-day mortality using the selected thresholds of A10_EXTEM_≤39 mm (NNS: 6, PPV: 0.32, NPV: 0.84) and A10_FIBTEM_<6 mm (NNS: 14, PPV: 0.25, NPV: 0.82).

The outcome was split into the time before and after the Rotem analysis. Before Rotem analysis, patients with A10_EXTEM_≤39 mm had more renal replacement therapy, required more coagulation factors, more blood transfusions, and a higher number of gastrointestinal bleedings occurred (Table 2). Interestingly, patients with A10_FIBTEM_<6 mm were comparable to patients with A10_FIBTEM_≥6 mm, only fibrinogen administration was significantly increased (Table 2).

**Fig. 2:**
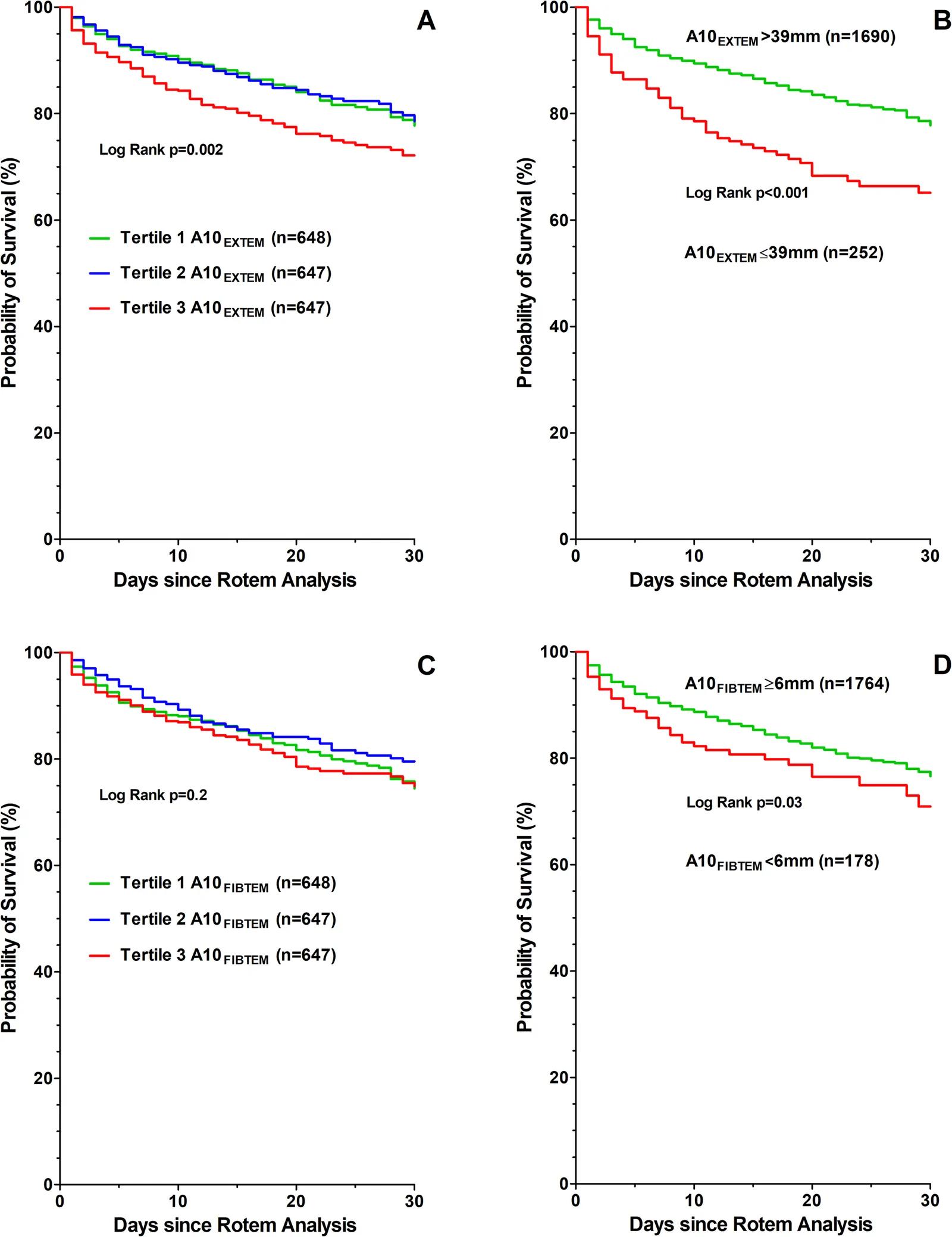
Kaplan-Meier survival plots for terciles of A10_EXTEM_ (**A**) and A10_FIBTEM_ (**C**). A10_EXTEM_>39 mm versus A10_EXTEM_≤39 mm during 30-day follow-up (**B**). A10_FIBTEM_≥6 mm versus A10_FIBTEM_<6 mm during 30-day follow-up (**D**). A10: amplitude of clot firmness 10 min after CT in mm. CT: clotting time. EXTEM: extrinsic pathway. FIBTEM: fibrin contribution to clot firmness. Rotem: Rotational thromboelastometry

Patients with A10_EXTEM_≤39 mm (A10_EXTEM_≤39 mm: 32% versus A10_EXTEM_>39 mm: 16%, *p* < 0.001) and A10_FIBTEM_<6 mm (A10_FIBTEM_<6 mm: 25% versus A10_FIBTEM_≥6 mm: 18%, *p* = 0.03; Table 2; Fig. 2) had an increased 30-day mortality.

Even after adjustment A10_EXTEM_≤39 mm, A10_EXTEM_, and A10_FIBTEM_<6 mm remained independent risk factors for 30-day mortality. This remained true in sensitivity analyses (Table 3). Interestingly, in all these multivariate models, CT_EXTEM_ (adjHR 1.002 [1.001–1.003], *p* < 0.001) and CT_INTEM_ (adjHR 1.001 [1.000–1.001], *p* = 0.002) remain independent risk factors for 30-day mortality.

**Table 3:** Cox proportional hazards models: A10FIBTEM and A10EXTEM a risk factor for 30-day mortality

	A10_EXTEM_≤39 mm		A10_EXTEM_		A10_FIBTEM_<6 mm		A10_FIBTEM_	
Model	HR (95% CI)	*p*-value	HR (95% CI)	*p*-value	HR (95% CI)	*p*-value	HR (95% CI)	*p*-value
crude in all patients (*n* = 1942)	2.1 (1.6–2.7)	***< 0.001***	0.98 (0.97–0.99)	***< 0.001***	1.4 (1.0–2.0)	***0.03***	1.00 (0.99–1.01)	*0.7*
Primary model in all patients (*n* = 1942)								
*adjusted all population characteristics*,* comorbidities*	1.7 (1.3–2.3)	***< 0.001***	0.98 (0.97–0.99)	***< 0.001***	1.6 (1.1–2.2)	***0.009***	0.99 (0.98–1.00)	*0.2*
Sensitivity Analyses in all patients (*n* = 1942)								
*adjusted all population characteristics*,* comorbidities*,* CT*_*INTEM*_, *CT*_*EXTEM*_	1.5 (1.1–1.9)	***0.009***	0.99 (0.98–0.99)	***0.002***	1.4 (1.0–1.9)	*0.06*	1.00 (0.98–1.01)	*0.3*
*adjusted all population characteristics*,* comorbidities*,* CT*_*INTEM*_, *CT*_*EXTEM*_ *and all variables before Rotem analysis from Table 2*	1.5 (1.1–2.0)	***0.007***	0.99 (0.98–0.99)	***0.002***	1.5 (1.1–2.1)	***0.02***	0.99 (0.98–1.01)	*0.3*

Primary model and sensitivity analyses. Data are expressed as HR (hazard ratio) with a 95% confidence interval (CI). Hazard ratio of A10_EXTEM_ and A10_FIBTEM_ in mm. Cox proportional hazard models were used to adjust for confounding factors. A10: amplitude of clot firmness 10 min after CT in mm. CT: clotting time in seconds (s). EXTEM: extrinsic pathway. FIBTEM: fibrin contribution to clot firmness. INTEM: intrinsic pathway without heparin neutralization. Rotem: Rotational thromboelastometry. All significant values are marked in bold

We compared survivors versus non-survivors in patients suffering from A10_EXTEM_≤39 mm and A10_FIBTEM_<6 mm. Interestingly, adverse events were quite comparable, whereas rotational thromboelastometry analysis showed impaired coagulation in non-survivors (Table 4). The increased mortality was mainly related to uncontrolled bleeding.

**Table 4:** 30-day mortality

	A10_EXTEM_≤39 mm					A10_FIBTEM_<6 mm				
	survivor		non-survivor		*p*-value	survivor		non-survivor		*p*-value
	**(** ***n*** ** = 172)**		**(** ***n*** ** = 80)**			**(** ***n*** ** = 134)**		**(** ***n*** ** = 44)**		
*Mech. ventilated (h)*	50	± 170	19	± 52	*0.1*	36	± 103	14	± 36	*0.2*
*Renal replacement therapy (%)*	30	(17)	27	(34)	***0.006***	15	(11)	14	(32)	***0.004***
*Length of stay (days)*										
*In ICU*	11	± 15	6	± 6	***0.002***	11	± 16	6	± 7	***0.04***
*In hospital*	27	± 28	6	± 7	***< 0.001***	26	± 28	7	± 8	***< 0.001***
*30-day mortality (%)*	0	(0)	80	(100)	***< 0.001***	0	(0)	44	(100)	***< 0.001***
*Packed red blood cells (n)*	3	± 5	3	± 4	*0.1*	3	± 3	3	± 4	*0.5*
*Platelet concentrate (n)*	2	± 2	1	± 1	***0.04***	1	± 2	1	± 2	*0.6*
*Fresh frozen plasma (n)*	5	± 75	1	± 2	*0.5*	1	± 2	1	± 2	*0.7*
*Prothrombin concentrate (IU)*	890	± 1966	813	± 1459	*0.8*	951	± 2034	761	± 1054	*0.6*
*Fibrinogen (g)*	2.3	± 3.6	1.8	± 2.8	*0.2*	2.8	± 4.0	2.2	± 2.8	*0.4*
*Recombinant Factor VIIa (IU)*	10	± 57	4	± 34	*0.4*	6	± 42	0	± 0	*0.3*
*Factor XIII (IU)*	80	± 409	31	± 280	*0.3*	84	± 411	57	± 377	*0.7*
*Adverse events*										
*Pneumonia (%)*	10	(6)	2	(3)	*0.3*	13	(10)	0	(0)	***0.04***
*Pulmonary embolism (%)*	1	(1)	1	(1)	*0.5*	0	(0)	1	(2)	*0.2*
*Acute myocardial infarction (%)*	0	(0)	2	(3)	*0.1*	0	(0)	1	(2)	*0.2*
*Embolic apoplexy (%)*	1	(1)	3	(4)	*0.1*	2	(1)	2	(5)	*0.3*
*Peripheral arterial embolism and thrombosis (%)*	1	(1)	0	(0)	*1*	0	(0)	0	(0)	─
*Gastrointestinal ischemia (%)*	1	(1)	3	(4)	*0.1*	2	(1)	2	(5)	*0.3*
*Gastrointestinal bleeding (%)*	3	(2)	2	(3)	*0.7*	1	(1)	1	(2)	*0.4*
*Rotational thromboelastometry*										
*CT EXTEM (s)*	108	± 130	139	± 176	*0.1*	94	± 43	165	± 223	***0.001***
*CT INTEM (s)*	246	± 158	326	± 225	***0.001***	229	± 101	324	± 210	***< 0.001***
*A10 EXTEM (mm)*	32	± 7	29	± 9	***0.02***	37	± 8	29	± 12	***< 0.001***
*A10 FIBTEM (mm)*	7.5	± 6.3	7	± 4.9	*0.5*	4.1	± 1.1	3.6	± 1.2	***0.01***
*CT APTEM (s)*	95	± 91	149	± 230	***0.008***	92	± 57	188	± 295	***< 0.001***
*A10 APTEM (mm)*	33	± 8	29	± 9	***< 0.001***	36	± 8	30	± 11	***< 0.001***

Survivor versus non-survivor in patients with A10_EXTEM_≤39 mm and A10_FIBTEM_<6 mm. Continuous variables are expressed as mean and standard deviation and were compared with a t-test. Categorical variables are presented as numbers (column percentages) and were compared with chi^2^ tests. A10: amplitude of clot firmness 10 min after CT in mm. CT: clotting time in seconds (s). APTEM: extrinsic pathway with inhibition of fibrinolysis. EXTEM: extrinsic pathway. FIBTEM: fibrin contribution to clot firmness. INTEM: intrinsic pathway without heparin neutralization. ICU: Intensive care unit. Packed red blood cells (450mL), Platelet concentrate (270mL), Fresh frozen plasma (300mL). All significant values are marked in bold

One therapeutic approach for patients with A10_FIBTEM_<6 mm is the administration of fibrinogen. Patients suffering from A10_FIBTEM_<6 mm were treated significantly more with fibrinogen before (A10_FIBTEM_<6 mm: 1.0 ± 3.8 g versus A10FIBTEM≥6 mm: 0.6 ± 2.5 g, *p* = 0.02) and after the Rotem analysis (A10_FIBTEM_<6 mm: 2.7 ± 3.7 g versus A10FIBTEM≥6 mm: 0.5 ± 1.6 g, *p* < 0.001; Table 2). The same was true for patients with A10_EXTEM_≤39 mm (Table 2).

In a larger population of 2103 patients, including 161 patients with missing information, the results were similar.

## Discussion

A10_EXTEM_≤39 mm, A10_EXTEM_ and A10_FIBTEM_<6 mm might be an independent risk factor for 30-day mortality, but A10_FIBTEM_ is not. The threshold, based on the study by Maegele et al., is A10_EXTEM_≤45 mm [6]. According to Weber et al. and Schmitt et al., it is A10_EXTEM_≤40 mm [2, 7]. The threshold of 39 mm obtained in this study is consistent with these consensus-based values, reinforcing its validity and clinical relevance. The threshold value A10_FIBTEM_<6 mm is based on previous studies on the detection of a relevant fibrinogen deficiency that required fibrinogen administration [2, 7–9]. A10_FIBTEM_<6 mm could be interpreted as a sign of massive blood loss. Overall mortality was 18%. This aligns with earlier findings in trauma patients experiencing massive bleeding [10, 11]. Although trauma-, surgery-, and obstetric-related coagulopathies arise from distinct and multifactorial pathophysiological mechanisms, they share common alterations in clot formation and stability that can be assessed using viscoelastic testing. As described by Görlinger et al., scenario-specific algorithms differ in diagnostic and therapeutic prioritization; however, Rotem parameters and their threshold values remain largely consistent, with differences mainly in their clinical interpretation [12]. They involve a disrupted balance between clot formation, breakdown, and natural anticoagulation, influenced by clot strength, platelet function, and interactions between coagulation factors. Contributing factors include blood loss, hemodilution, platelet function, coagulation factor consumption, activation of fibrinolysis and inflammation, and hypothermia [9, 13]. Early mortality in bleeding is often driven by ongoing hemorrhagic shock, trauma-induced coagulopathy, and incomplete resuscitation. Known risk factors for complications and death include age, acute kidney or lung injury, emergency presentation, coagulation disorders, and gastrointestinal ischemia [11, 14–18].

It is noteworthy that non-survivors with an A10_EXTEM_≤39 mm and A10_FIBTEM_<6 mm showed significantly worse results in the rotational thromboelastometry analysis, while adverse events were almost similar (Table 4).

Nevertheless, to minimize the influence of adverse events as potential confounders, outcomes were analyzed separately for the periods before and after rotational thromboelastographic assessment (Table 2). Adverse effects occurring prior to rotational thromboelastography and rotational thromboelastometry analysis were included as covariates in the sensitivity analysis. Despite these adjustments, an A10_EXTEM_≤39 mm and an A10_FIBTEM_<6 mm remained an independent predictor of 30-day mortality.

This finding suggests that mortality in these patients may be primarily driven by underlying hemostatic failure in the extrinsic and intrinsic pathway, particularly affecting clot formation and stabilization. Hemostasis occurs in three coordinated phases to prevent and control bleeding.

Primary hemostasis is initiated by vasoconstriction, followed by platelet activation leading to the formation of a temporary platelet plug [19]. Impaired platelet function increases the risk of excessive bleeding. Disruption of platelet activity predisposes individuals to an increased bleeding tendency. Platelet dysfunction can be inherited, resulting from abnormalities in platelet structure or function, or acquired due to medications, underlying systemic conditions, or medical interventions [9, 20, 21]. The clinical presentation ranges from the absence of symptoms to severe and potentially life-threatening bleeding events [20, 21].

Secondary hemostasis is when the coagulation cascade is activated [19]. The common pathway begins when the intrinsic or extrinsic system activates factor X (Stuart-PowerFactor) to factor Xa. Once factor Xa is generated, it combines with cofactor V (proaccelerin) to convert prothrombin (factor II) into thrombin (factor IIa). Thrombin then plays a central role by converting fibrinogen into fibrin. Early polymerization occurs within minutes and takes up to hours. Activation of factor XIII (fibrin-stabilizing factor) to cross-link fibrin strands increases clot stiffness, resistance to deformation, and degradation. This happens on a longer time scale (hours). This fibrin mesh stabilized the platelet plug, completing the clotting process and preventing further bleeding.

Inherited deficiencies of factors involved in the common pathway – factor II, III, V, X, and XIII – are very rare, with symptoms ranging from asymptomatic cases to severe bleeding manifestations [22–25].

Finally, tertiary hemostasis involves the breakdown of the fibrin clot through plasminogen activation, allowing for controlled clot resolution once vessel integrity is restored [19]. Increased fibrinolysis, or hyperfibrinolysis, can result from various clinical conditions such as severe trauma, major surgery, liver disease, and sepsis [26–28]. Hypotension is a known risk factor for hyperfibrinolysis as well [28]. These states often trigger excessive activation of tissue plasminogen activator or impair natural inhibitors like alpha-2-antiplasmin, leading to unregulated fibrin breakdown and bleeding. Increased fibrinolysis is treated with tranexamic acid to prevent fibrin breakdown and fibrinogen replacement if levels are low.

In Europe, the initial management of coagulation disorders during massive bleeding typically involves the administration of coagulation factor concentrates and has proven to reduce blood products and mortality [7, 9, 29]. At our hospital, coagulation management during bleeding was guided by the goal-directed rotational Rotem-based approach recommended by Weber et al. [2].

The abnormal parameter of A10_EXTEM_ less than 40 mm with a normal FIBTEM during bleeding indicates reduced platelet function or count and suggests the need for platelet concentrate transfusion or desmopressin [2, 7]. All adult patients received a minimum of 1 g tranexamic acid, and pediatric patients received 30 mg/kg body weight. Platelet concentrates were administered as indicated, and desmopressin was considered in patients with known platelet dysfunction. After an initial treatment of the primary hemostasis during bleeding with coagulation factor concentrates, the addition of fibrinogen should be considered, especially when A10_FIBTEM_ is lower than 10 mm [2, 7, 9]. Often the first factor to decrease during massive bleeding is fibrinogen, and should be replaced – usually in combination with tranexamic acid [30, 31]. Platelet concentrates were administered as indicated, and desmopressin was considered in selected cases but not routinely given. Fibrinogen not only enhances clot firmness, but it also optimizes coagulation, reduces bleeding by 32%, and significantly decreases transfusion requirements [32–35]. The association between fibrinogen use and lower survival probably reflects that fibrinogen was predominantly administered to patients with greater hemostatic impairment. Rather than indicating a harmful effect of the treatment.

When interpreting A10_FIBTEM_, it should be considered that this parameter represents a single, readily correctable measurement with administration of fibrinogen. This could explain why A10_FIBTEM_ was not identified as a risk factor for mortality, in contrast to A10_EXTEM_, CT_EXTEM_ and CT_INTEM,_ which may be influenced by the absence of optimized or targeted therapy. In comparison, CT_EXTEM_ and CT_INTEM_ reflect more complex and integrated aspects of the coagulation and, therefore, are more strongly associated with adverse outcomes [4, 5].

### Limitations

While potential causes of clotting weakness and corresponding treatment strategies are well established, our study does not allow us to determine whether the observed findings in our cohort reflect insufficient prior therapy or an independent coagulopathic state. Furthermore, we did not assess the effect of therapeutic interventions on these Rotem parameters. We did not include MCF (maximum clot firmness) or A5 (the clot firmness at 5 min) in our study. However, in a study by Schoch et al., both A10_FIBTEM_ and MCF demonstrated comparable ability to conventional laboratory parameters in predicting the need for massive transfusion in trauma patients [36]. Notably, A10 showed a stronger association with platelet count and fibrinogen levels, indicating that it may better reflect the dynamic aspects of hemostasis than MCF [37].

There are no reliable data on average blood loss, and severity of thrombocytopenia, data on hypocalcemia, acidosis, and hypothermia were also not available. Nevertheless, these latter parameters were managed according to the Weber et al. guidelines as well as treatment for specific coagulation disorder [2]. Another limitation of our study is that it was conducted within a single patient population. Validation in a larger multicenter cohort is warranted, and a prospective cohort study would help overcome these limitations through real-time data collection.

## Conclusion

In our study, A10_EXTEM_ ≤39 mm and A10_FIBTEM_ <6 mm were associated with 30-day mortality, whereas no such association was observed for A10_FIBTEM_ alone. While reduced A10_FIBTEM_ may indicate the need for fibrinogen supplementation in bleeding patients, Rotem parameters, including A10_EXTEM_, CT_EXTEM_ (extrinsic pathway), and CT_INTEM_ (intrinsic pathway), should be interpreted within the overall clinical context and may support individualized coagulation management.

## Supplementary Information

Below is the link to the electronic supplementary material.


Supplementary Material 1 (DOCX 27.7 KB)


## Data Availability

The data that support the findings of this study are available from the authors on reasonable request.
